# Cyberchondria-Related Behaviors Among Undergraduate Female Healthcare Students: A Descriptive and Exploratory Study of CSS-12 Dimensions and Demographic Characteristics

**DOI:** 10.3390/healthcare14193185

**Published:** 2026-09-26

**Authors:** Wafa Hamad Almegewly, Maryam Soliman Almalki, Lama Mohammed Alanazi, Wajd Khalid Alsaud, Shahad Saad Alshammari, Rawabi Fahad Alfraig, Ghala Taresh Alotaibi, Dalal Yahya Alqarni, Bayan Abdulrahman Arishi, Nada Saeed Alshahrani, Abdelaziz Hendy, Wireen Leila Dator, Rasha Mohammed Hussien

**Affiliations:** 1 Department of Community and Psychiatric Mental Health Nursing, College of Nursing, Princess Nourah bint Abdulrahman University, Riyadh 11671, Saudi Arabia; 2 College of Nursing, Princess Nourah bint Abdulrahman University, Riyadh 11671, Saudi Arabia; 443005654@pnu.edu.sa (M.S.A.); 443005443@pnu.edu.sa (L.M.A.); 443005750@pnu.edu.sa (W.K.A.); 443005524@pnu.edu.sa (S.S.A.); 443007316@pnu.edu.sa (R.F.A.); 443005704@pnu.edu.sa (G.T.A.); 443005769@pnu.edu.sa (D.Y.A.); 443005658@pnu.edu.sa (B.A.A.); 443007036@pnu.edu.sa (N.S.A.); 3 Department of Maternal and Child Health Nursing, College of Nursing, Qassim University, Buraydah 52571, Saudi Arabia; a.hendy@qu.edu.sa; 4 College of Nursing and Allied Health Sciences, St. Paul University Manila, Manila 1004, Philippines; sweetziyi2002@gmail.com; 5 Department of Community, Psychiatric, and Mental Health Nursing, College of Nursing, Qassim University, Buraydah 52571, Saudi Arabia; rm.ahmed@qu.edu.sa

**Keywords:** cyberchondria, healthcare students, cross-sectional study, Saudi Arabia, digital health behavior, health anxiety

## Abstract

**Background**: Cyberchondria, characterized by excessive or repeated online searching for health-related information accompanied by heightened health concerns or distress, is an increasingly recognized behavioral phenomenon. Healthcare students represent an important population for investigation due to their frequent exposure to medical information and digital health resources. However, the extent of cyberchondria-related behaviors and their relationship with demographic and academic characteristics remain insufficiently explored in this population. Objectives: This study aimed to describe the level and distribution of cyberchondria-related behaviors and to explore whether selected demographic and academic characteristics were associated with CSS-12 scores among undergraduate female healthcare students from different academic programs at a Saudi university. **Methods**: A cross-sectional descriptive correlational design was employed. A proportionate stratified non-probability convenience sample of 362 female students was recruited from six health-related colleges. Data were collected using the validated Cyberchondria Severity Scale (CSS-12), which measures four dimensions: excessiveness, distress, reassurance, and compulsion. Descriptive statistics, Pearson correlation, and multiple linear regression were applied using SPSS version 29. **Results**: The overall CSS-12 mean score indicated a moderate level of cyberchondria-related behaviors (M = 32.46, SD = 5.80). At the dimensional level, Excessiveness recorded the highest mean score (M = 10.29, SD = 2.10), followed by Distress (M = 7.46, SD = 2.35), while Reassurance (M = 7.73, SD = 2.20) and Compulsion (M = 6.98, SD = 2.05) showed comparatively lower scores. Based on the sample-derived score distribution, 23.8% of participants were classified in the upper-score group, while 30.4% and 45.9% were classified in the middle- and lower-score groups, respectively. Correlation analysis revealed significant positive relationships among all CSS-12 dimensions (*p* < 0.001), with correlation coefficients ranging from weak to strong (r = 0.31–0.56). Multiple linear regression analysis showed that age, GPA, academic year, and academic program were not significantly associated with CSS-12 scores (*p* > 0.05), and the model explained a very small proportion of variance (R^2^ = 0.006), indicating limited explanatory power. **Conclusions**: Cyberchondria-related behaviors were distributed across low, moderate, and high score categories among female healthcare students, with excessiveness emerging as the most prominent dimension. The findings demonstrate interrelationships among CSS-12 dimensions but no significant associations with demographic or academic variables. Overall, the results are descriptive and correlational in nature and indicate that the demographic and academic characteristics examined in this study did not meaningfully account for variation in cyberchondria-related behaviors. Psychological and behavioral factors not assessed in the present study warrant investigation in future research.

## 1. Introduction

The rapid expansion of internet use and the increasing availability of online health information have contributed to growing interest in cyberchondria as an emerging health-related behavioral phenomenon [1]. Cyberchondria is generally described as excessive or repeated searching for health-related information online that may be associated with increased health concerns, anxiety, or emotional distress [2]. It is considered a multidimensional construct comprising four interrelated components: excessiveness, reflecting frequent and repeated online health information searching; compulsion, representing difficulty controlling searching behaviors; distress, referring to emotional responses such as worry or fear following online searches; and reassurance seeking, involving repeated attempts to obtain confirmation or reduce uncertainty through online or professional health information sources [3].

The COVID-19 pandemic further increased dependence on digital health information due to restricted healthcare access, heightened uncertainty, and widespread circulation of health-related information and misinformation [4]. Within healthcare education, students represent a unique population of interest because they are frequently exposed to medical terminology, disease-related information, and health sciences content as part of their academic training [5,6,7]. The Internet has also become an increasingly important and accessible source of health information and health-related services [8]. Such exposure and accessibility may be associated with patterns of online health information seeking; however, the specific psychological mechanisms underlying these behaviors require further investigation.

Cyberchondria has been investigated across diverse populations, including the general public, healthcare professionals, university students, and professionals working in other occupational sectors [9]. However, direct comparisons of reported levels across studies should be interpreted cautiously due to differences in measurement instruments, scoring approaches, sample characteristics, and the absence of universally accepted clinical cut-off points. Therefore, reported categories and percentages represent study-specific score distributions rather than epidemiological prevalence estimates.

Previous studies have demonstrated that cyberchondria-related behaviors occur across different cultural and occupational groups [9]. During and following the pandemic period, studies from countries including India and Nigeria reported moderate-to-high levels of cyberchondria-related behaviors among different populations [10,11]. In Saudi Arabia, elevated cyberchondria-related behaviors have been reported among adults, with associations between online health searching, somatic symptom reporting, and health-related concerns, particularly among younger individuals [12]. Additionally, online self-diagnosis behaviors have been described as a common pattern associated with the accessibility and convenience of digital health information [12,13].

The existing literature suggests that cyberchondria may involve complex interactions between behavioral, psychological, and digital information-seeking factors. Previous research has examined its relationship with constructs such as health anxiety, intolerance of uncertainty, obsessive–compulsive tendencies, internet-related problematic behaviors, and approaches to evaluating online health information [13,14,15]. However, these psychological and behavioral constructs are not assessed in the present study and are considered important areas for future investigation rather than variables examined within the current analysis.

Within healthcare education, online health resources represent an important component of learning and professional development [16]. Nevertheless, studies have reported varying levels of cyberchondria-related behaviors among healthcare students across different disciplines. Nursing students in Egypt, Jordan, and Iran have demonstrated notable levels of cyberchondria-related behaviors [5,17,18]. Similarly, studies among medical students have reported moderate to high levels, with variations observed across different educational settings [19]. Dental students have also demonstrated increased cyberchondria-related behaviors during the COVID-19 pandemic period [20].

Several demographic and academic characteristics have been explored as potential correlates of cyberchondria-related behaviors. Previous studies have examined variables such as age, academic year, academic performance, and gender, with inconsistent findings across populations. Some studies have reported higher levels among younger students, students with higher academic achievement, or those in earlier academic years, whereas others have reported different patterns [6,7,18,21]. These variations suggest that demographic and academic characteristics alone may not fully explain differences in cyberchondria-related behaviors across populations.

Despite increasing research interest, important gaps remain. Limited evidence is available regarding cyberchondria-related behaviors among all-female healthcare student populations, particularly within Middle Eastern educational contexts. Furthermore, few studies have examined differences across multiple healthcare disciplines within the same institutional environment, such as in Saudi Arabia, where internet and smartphone use are widespread, to better characterize cyberchondria-related behaviors among future healthcare professionals.

Therefore, this study aimed to assess the level and distribution of cyberchondria-related behaviors and explore whether selected demographic and academic characteristics were associated with CSS-12 scores among undergraduate female healthcare students enrolled in different academic programs at a Saudi university.

Understanding cyberchondria-related behaviors within this specific population is important, as healthcare students represent future healthcare professionals who are frequently exposed to medical information and digital health resources. Examining these behaviors among healthcare students may show how online health information-seeking patterns manifest within an academically health-focused environment. Although the inclusion of an all-female academic setting limits generalizability to other populations, it provides a context-specific understanding of cyberchondria-related behaviors within this educational setting.

The following research questions were addressed:

- What is the level and distribution of cyberchondria-related behaviors among female healthcare students in an all-female university setting?
- Are selected demographic and academic characteristics associated with variations in CSS-12 scores among female healthcare students?

The conceptual background of this study is informed by previous literature suggesting that cyberchondria is a multifactorial behavioral phenomenon that may be associated with demographic, academic, psychological, and digital information-seeking characteristics. Demographic and academic factors, including age, academic year, academic performance, and academic program, have been examined in previous studies as potential correlates of cyberchondria-related behaviors, although findings have been inconsistent across populations and educational settings [22,23,24,25].

Differences across academic groups may reflect variations in educational experiences, exposure to health-related information, and learning environments; however, these underlying mechanisms were not directly assessed in the present study. Similarly, psychological and cognitive–behavioral constructs, including health anxiety, uncertainty-related processes, and health information evaluation patterns, have been proposed in previous literature as important contributors to cyberchondria but were beyond the scope of the current analysis.

Accordingly, the present study focused on exploring whether selected demographic and academic characteristics were associated with CSS-12 scores among female healthcare students while recognizing that a more comprehensive understanding of cyberchondria requires future investigation of psychological and digital-behavioral factors.

## 2. Materials and Methods

### 2.1. Study Design and Setting

This study employed a cross-sectional descriptive correlational design. This design is appropriate for describing the level of cyberchondria and examining statistical associations between cyberchondria scores and selected demographic and academic variables at a single point in time. However, it does not allow for establishing causality, directionality, or temporal changes over time. The study was conductedat Princess Nourah bint Abdulrahman University, Riyadh, Saudi Arabia, across six health-related academic areas: the College of Nursing, the College of Medicine, the College of Dentistry, the College of Pharmacy, the College of Health and Rehabilitation, and the College of Foundation Health Year. The foundation year program in this university provides the common preparatory requirements that include foundational knowledge in sciences and academic skills for the health degrees. The university is an institution that provides education exclusively for women; hence, the study participants were all female.

### 2.2. Population and Sampling

The target population comprised 6015 female undergraduate students enrolled in health-related academic programs at the participating university. The population was distributed across six health-related academic areas: Nursing (*n* = 1623), Medicine (*n* = 470), Health and Rehabilitation Sciences (*n* = 2314), Pharmacy (*n* = 340), Dentistry (*n* = 175), and the Foundation Health Year program (*n* = 1093), which was considered an academic program rather than a college.

Participants were recruited using a proportionate stratified non-probability convenience sampling approach. Each health-related academic program constituted a separate stratum, and the target sample allocation for each stratum was determined according to its proportional representation within the total eligible student population. Within each stratum, eligible students were approached through official university email and the administrative communication channels of their respective colleges or programs. The electronic survey link was distributed through these channels, and participation was voluntary and based on electronic informed consent. Recruitment proceeded within the predefined proportional allocation for each stratum until the required sample size was achieved.

A total of 407 students were invited to participate, of whom 368 initially completed the survey, yielding an initial response rate of 90.4%. Following data screening, six responses were excluded because of incompleteness or duplication, resulting in a final analytic sample of 362 participants. Reminder messages were sent three times during the data collection period to enhance participation, and no incentives were provided. The final recruitment strategy therefore maintained proportional representation across the predefined academic strata while using convenience-based participation within each stratum.

### 2.3. Sample Size

The sample size was initially determined based on the primary descriptive objective of the study using the Raosoft sample size calculator (Raosoft, Inc., Seattle, WA, USA). Assuming a 95% confidence level, a 5% margin of error, and a total population of 6015 eligible students, the minimum required sample size was estimated to be 362 participants.

Following determination of the required sample size, proportionate stratified non-probability convenience sampling was applied to reflect the relative distribution of students across the participating health-related academic programs. Each college or academic program constituted a separate stratum. Based on the population distribution, the proportional allocation was 26.8% for Nursing, 7.7% for Medicine, 38.4% for Health and Rehabilitation Sciences, 5.5% for Pharmacy, 3.3% for Dentistry, and 18.3% for the Foundation Year program. Accordingly, the final sample comprised 97 Nursing students, 28 Medicine students, 139 Health and Rehabilitation Sciences students, 20 Pharmacy students, 12 Dentistry students, and 66 Foundation Year students. These allocations were intended to preserve the relative representation of the academic programs within the participating university and should not be interpreted as probability sampling within each stratum.

Because the original sample size calculation was designed primarily for the descriptive component of the study rather than specifically for multiple linear regression, a sensitivity power analysis was additionally conducted for the exploratory regression model. With a final sample size of 362 participants, four independent variables (age, GPA, academic year, and academic program), a significance level of α = 0.05, and 80% statistical power, the analysis indicated that the study was capable of detecting an overall effect size of approximately Cohen’s f^2^ = 0.033, corresponding to an R^2^ of approximately 0.032. Thus, the available sample provided adequate power to detect small overall effects explaining approximately 3.2% or more of the variance, whereas smaller multivariable associations may not have been detected reliably. Accordingly, the regression analysis was considered exploratory, and its findings were interpreted cautiously with attention to effect estimates, confidence intervals, statistical significance, and explained variance.

### 2.4. Inclusion/Exclusion Criteria

Inclusion criteria for study participation were: (1) students should be enrolled in one of the undergraduate health programs at the selected university and (2) should be capable of giving informed consent. Incomplete and duplicate responses were also excluded during the process to ensure data quality and reliability.

### 2.5. Data Collection Instruments

The data were collected using the Cyberchondria Severity Scale (CSS-12) [3], a well-established and commonly used instrument. The CSS-12 is a short adaptation of the original CSS and has shown strong psychometric properties. Confirmatory Factor Analysis (CFA) performed by the tool developers established the overall internal consistency values of both the total scale and subscales, which were acceptable to excellent [3]. The reliability testing done in this study by the researchers yielded a Cronbach’s alpha of 0.90, indicating excellent internal consistency. The four subscales were likewise found to have satisfactory to good internal consistency, ranging from α = 0.73 to 0.87.

The CSS-12 consists of 12 items rated on a 5-point Likert scale (1 = never to 5 = always), with total scores ranging from 12 to 60. For descriptive analytical purposes, total scores were categorized into sample-derived lower-, middle-, and upper-score groups based on the observed score distribution. Sample-specific tertile cut-points corresponding to the 33.3rd and 66.7th percentiles were used to define the three score categories. Because the CSS-12 total score is discrete and participants with identical scores at the percentile boundaries were retained within the same category, the resulting groups were not necessarily equal in size. These categories were used solely for descriptive classification and do not represent clinically validated cut-off points or diagnostic thresholds [5].

The four dimensions include the following: Excessiveness: Indicates repeated and recurring online queries for health information. Distress: Aspects of anxiety and emotional distress induced by online searches. Reassurance: Shapes the propensity Compulsion: Assesses the extent to which online searching disturbs life or study. In addition to the CSS-12, a brief sociodemographic questionnaire was incorporated to collect information on age, gender, academic program, academic year, and self-reported GPA. The questionnaire was provided in English. Arabic translation was not necessary since the students can speak and understand the English language, which is also the official language of instruction across all the colleges.

### 2.6. Data Collection Procedure

Data were collected over a two-week period using an electronic questionnaire administered through Google Forms. Eligible students were invited through official university email and college administrative communication channels. The survey link was distributed separately within the participating academic strata according to the planned recruitment framework. Participation was voluntary and anonymous, and no incentives were offered. Reminder messages were sent during the data collection period to improve response rates.

To limit repeated entries to the Google survey, management settings were enabled, and additional data quality checks were completed in the data screening process. Duplicate responses were determined by comparing survey data, response patterns, timestamps, and survey participant IP addresses. The researchers retained no duplicate survey responses. Research data consisted of survey responses that were not left blank. The survey response that contained the fewest number of blank responses to the critical survey items, the CSS-12 survey items, and the survey demographic items was retained. In this study, survey responses were considered complete if no items on the survey were left blank.

Survey responses were downloaded from Google Forms to conduct the first iteration of data management in Microsoft Excel. Completed data sets were downloaded to IBM SPSS Statistics version 29 for the remaining data management and analysis. Data quality checks were conducted to ensure no data were left blank. The CSS-12 survey response data sets were checked to ensure response data were not greater than the 1–5 response range of the Likert scale. Demographic data were checked for logical consistency. Three hundred sixty-two responses were deemed complete and met the study criteria and were retained for data analysis.

### 2.7. Ethical Considerations

This study was conducted in accordance with ethical research standards. Approval was obtained from the Institutional Review Board (IRB No. 24-0751). Electronic informed consent was secured from all participants prior to participation. Participation was entirely voluntary, and respondents were informed of their right to withdraw at any time without penalty. No personally identifiable information was collected, ensuring anonymity and confidentiality. All data were securely stored and used exclusively for research purposes.

### 2.8. Data Analysis

Data were analyzed using IBM SPSS Statistics (version 29) (IBM Corp., Armonk, NY, USA). Descriptive statistics were computed for all variables, including means, standard deviations, frequencies, and percentages as appropriate. The normality of continuous variables was assessed prior to inferential analyses.

The CSS-12 total score was calculated as the sum of all 12 items, ranging from 12 to 60, and was treated as the primary outcome variable in all inferential analyses. Subscale scores (Excessiveness, Distress, Reassurance, and Compulsion) were computed by summing their corresponding items according to the original validated structure of the instrument. Item-level mean scores were also calculated for descriptive purposes only. For descriptive classification, CSS-12 total scores were categorized into low, moderate, and high groups using a tertile-based approach based on the distribution of the study sample. These categories were used strictly for descriptive interpretation and do not represent clinically validated cut-off points or diagnostic thresholds.

Prior to regression analysis, all independent variables were carefully coded and recategorized where necessary to ensure statistical adequacy and model stability. Age was grouped into three categories (18–20, 21–23, and ≥24 years); however, due to numbers in the oldest category, it was combined with adjacent groups for regression analysis to avoid sparse data bias. GPA was treated as an ordinal categorical variable reflecting its structured grading system. Academic year was recoded to combine higher academic levels (fifth year, sixth year, and interns) due to small subgroup sizes. The academic program was collapsed into two categories (foundation year and specialized health programs) to ensure sufficient statistical power across groups. Academic program and academic year were recategorized prior to regression analysis to reduce sparse-data problems and improve the stability of the model estimates. Academic program was collapsed into two broader categories: Foundation Year and Specialized Health Programs, with the latter including Nursing, Medicine, Dentistry, Pharmacy, and Health and Rehabilitation Sciences. This grouping was necessary because several individual programs had relatively small subgroup sizes, which could have produced unstable regression estimates. Similarly, academic year was recategorized into broader training-level groups because of the small numbers of participants in the fifth year, sixth year, and internship categories. These recategorizations were undertaken for statistical stability; however, the resulting categories represent broad analytical groupings and should not be interpreted as indicating equivalence among the original academic disciplines or training levels.

Multiple linear regression analysis was performed to examine the association between demographic and academic variables and the total CSS-12 score. The assumptions of regression analysis, including multicollinearity, were tested using the Variance Inflation Factor (VIF), with values indicating no evidence of multicollinearity. Regression coefficients (B), standard errors (SE), standardized beta values (β), t-statistics, *p*-values, and 95% confidence intervals were reported. The level of statistical significance was set at *p* < 0.05. The multiple linear regression analysis was considered exploratory, as the original sample size calculation was not specifically based on a priori power requirements for multivariable modeling.

All statistical tests were two-tailed. Results were interpreted cautiously, with emphasis on effect sizes and explained variance in addition to statistical significance, in order to avoid over-interpretation of weak models. Normality assessment showed that the total cyberchondria score did not significantly deviate from normality (Shapiro–Wilk W = 0.98, *p* = 0.073). Skewness and kurtosis values ranged between −0.42 and 0.38 and −0.71 to 0.65, respectively, remaining within acceptable thresholds (±1). Visual inspection of histograms and Q–Q plots further supported the approximate normal distribution of the study variables. Accordingly, parametric statistical tests, including Pearson’s correlation and multiple linear regression, were considered appropriate for the analysis. Statistical significance was set at *p* < 0.05.

## 3. Results

The sample comprised 362 participants with a predominance of younger age groups, as most students were aged 18–20 years (59.7%), followed by 21–23 years (39.5%). Only a very small proportion were aged 24 years and above. Regarding academic programs, the largest proportion of participants was from Health Rehabilitation Sciences (38.4%), followed by Nursing (26.8%) and Foundation Year (18.3%), while smaller proportions were from medicine, pharmacy, and dentistry. In terms of academic level, first-year students represented the highest percentage (30.7%), and interns constituted the smallest group (3.9%). For GPA, most participants reported high academic achievement, with 43.7% scoring 4.1–4.5 and 37.8% scoring 4.6–5.0, while only a minimal proportion reported lower GPA ranges; see Table 1.

Item-level analysis showed variability in cyberchondria-related behaviors across different statements. The highest mean scores were observed for searching the internet when experiencing unexplained bodily sensations (M = 3.83, SD = 1.30) and reading multiple web pages about the same condition (M = 3.66, SD = 0.81).

Moderate scores were observed for repeated symptom searching (M = 2.81, SD = 1.12), distraction during online activities (M = 2.79, SD = 1.10), panic after reading about serious conditions (M = 2.90, SD = 1.05), and consulting a general practitioner (M = 2.90, SD = 1.04).

Lower scores were observed for interference with work/study (M = 2.22, SD = 0.83), interference with social activities (M = 1.97, SD = 0.64), emotional distress after searching (M = 1.97, SD = 0.78), and consultation with specialists (M = 2.58, SD = 1.17). Full item-level results are presented in Table 2.

As shown in Table 3, the excessiveness dimension recorded the highest mean score (M = 10.29, SD = 2.10). Distress followed with a comparatively lower mean score (M = 7.46, SD = 2.35). Reassurance (M = 7.73, SD = 2.20) and Compulsion (M = 6.98, SD = 2.05) showed lower mean scores within the sample. The overall CSS-12 total score was 32.46 (SD = 5.80). In terms of the sample-derived tertile-based score distribution, 23.8% of participants were classified in the upper-score group, while 30.4% and 45.9% were classified in the middle- and lower-score groups, respectively.

Pearson correlation analysis showed significant positive associations among all CSS-12 dimensions (*p* < 0.001). Excessiveness was positively correlated with distress (r = 0.42), reassurance (r = 0.31), and compulsion (r = 0.45). Distress showed significant associations with reassurance (r = 0.39) and compulsion (r = 0.56), while reassurance was also strongly correlated with compulsion (r = 0.52). Overall, all dimensions demonstrated moderate to strong interrelationships; see Table 4.

The multiple linear regression model was not statistically significant and demonstrated negligible explanatory power (R^2^ = 0.006; adjusted R^2^ = −0.005). None of the examined demographic and academic variables, including age, GPA, academic year, or academic program, was significantly associated with CSS-12 scores. Given the minimal variance explained by the model, these findings were interpreted cautiously and were not considered predictive (Table 5).

## 4. Discussion

### 4.1. Key Findings of the Study

The present study examined cyberchondria-related behaviors among female undergraduate students enrolled in health science programs. The findings indicated that cyberchondria-related behaviors were distributed at a moderate level based on the CSS-12 total score. Among the four dimensions, excessiveness was the most prominent, followed by distress, while reassurance and compulsion showed comparatively lower mean scores. This pattern suggests that repetitive online health information searching is the most dominant behavioral feature within the studied population, whereas interference with daily functioning and reassurance-seeking behaviors appear less prominent.

At the item level, students most frequently reported searching the internet when experiencing unexplained bodily sensations and consulting multiple online sources for the same condition. In contrast, lower scores were observed for items related to functional impairment and emotional distress after online searching.

### 4.2. Comparison with Previous Studies

The findings of the present study are broadly consistent with previous research conducted in Saudi Arabia and Egypt, which reported similar patterns of cyberchondria-related behaviors among healthcare students [5,26]. The similarity of findings across these studies suggests that comparable patterns of cyberchondria-related behaviors may occur in some healthcare student populations; however, differences in study populations, educational settings, and measurement approaches should be considered, and the underlying mechanisms require further investigation.

Previous studies have reported higher distress-related cyberchondria dimensions among students in earlier academic years, with authors suggesting that differences in clinical exposure and confidence in symptom interpretation may contribute to these variations [7]. However, contrasting findings have also been reported, with some studies observing higher levels among senior students or those with higher academic achievement [18]. These inconsistencies across studies may reflect differences in academic structures, curricula, student characteristics, cultural contexts, and measurement approaches [27].

### 4.3. Interpretation of Subscale Patterns (Literature-Based)

The finding that excessiveness was the most prevalent construct in this research is consistent with preceding discoveries amongst medical students, where repeated online searching is described as a main characteristic of cyberchondria [28]. As the second-most prevalent construct, distress was similar to the research that shows that searching for health-related information on the internet may elicit emotions such as anxiety and worry about the presence of expected symptoms [1]. Conversely, the levels of reassurance seeking in this research were found to be low to an extent, which was disparate to findings from some populations of university students who were not affiliated with university courses that were medically related, among whom the reassurance-seeking behavior was higher in frequency [29]. One possible interpretation is that differences in health-related knowledge between healthcare students and students from non-health disciplines may contribute to variations in reassurance-seeking behavior; however, this explanation was not directly examined in the present study and should therefore be considered tentative [7].

### 4.4. Exploratory Regression Analysis

The regression analysis provided limited explanatory value. The model was non-significant, explained only 0.6% of the variance in CSS-12 scores, and none of the included demographic and academic variables reached statistical significance. Therefore, the regression findings should be regarded as exploratory and should not be overinterpreted. Rather than suggesting that demographic and academic characteristics are unimportant, the results indicate only that the variables included in the present model did not meaningfully explain variation in CSS-12 scores within this sample. Future studies should use more theoretically driven models and include additional variables that may better account for individual differences in cyberchondria-related behavior.

Given the exploratory nature of the model, the regression analysis was intended to assess basic associations rather than establish predictive relationships. The limited explanatory capacity of the model indicates that the demographic and academic variables examined in this study accounted for very little of the observed variation in CSS-12 scores. Although the sensitivity analysis indicated that the available sample was sufficient to detect overall effects explaining approximately 3.2% or more of the variance, smaller multivariable associations may have remained undetected. Therefore, the non-significant regression findings should be interpreted cautiously and should not be taken as evidence of the complete absence of associations between demographic or academic characteristics and CSS-12 scores.

### 4.5. Future Research Directions

Psychological, cognitive, and digital-behavioral factors such as health anxiety, intolerance of uncertainty, obsessive–compulsive symptoms, eHealth literacy, problematic internet use, and somatic symptom perception have been discussed in previous literature in relation to cyberchondria-related behaviors [30,31]. However, these factors were not assessed in the present study and therefore cannot be used to explain the observed findings. Their potential contribution should be considered a hypothesis for future research rather than an explanatory mechanism supported by the current data. Future studies should directly assess these constructs using theoretically informed multivariable models and, where feasible, longitudinal designs to clarify their associations with cyberchondria-related behaviors and their patterns over time.

### 4.6. Study Implications

The results emphasize the need for better knowledge and skills in terms of health awareness and web usage. Healthcare students should be prompted and taught more about how health-related information is to be searched, extracted, and critically analyzed.

Taken together, the findings of this study contribute to the growing body of literature on cyberchondria by providing empirical evidence from an underrepresented population of female healthcare students in a Middle Eastern context. Within the present sample, the observed pattern of CSS-12 scores, characterized by relatively higher excessiveness and distress scores than reassurance and compulsion scores, provides a descriptive picture of online health information-seeking tendencies among the participating female healthcare students.

Importantly, the absence of significant associations between demographic–academic variables and cyberchondria scores, along with the very limited explanatory power of the regression model, adds to the literature by suggesting that such variables alone are insufficient to explain variability in cyberchondria. This supports a shift toward more psychologically driven explanatory models in future research.

By providing a detailed dimensional analysis of CSS-12 and clarifying its scoring structure in a non-clinical population, this study also contributes methodologically to the interpretation and application of the scale in similar educational contexts. These findings were collectively more comprehensive, theory-driven models that incorporate psychological and digital behavior factors to better understand cyberchondria in healthcare student populations.

### 4.7. Strengths

This study has several noteworthy strengths. First, it addresses an important and underexplored area by examining cyberchondria among female healthcare students in a Middle Eastern context, contributing to the limited regional literature on this topic. Second, the study includes participants from six different health-related academic programs, enhancing the breadth of representation within the healthcare education context. Third, the use of the validated Cyberchondria Severity Scale (CSS-12). Fourth, the analysis distinguishes between multiple dimensions of cyberchondria, providing a more nuanced understanding of online health-related behaviors rather than relying solely on total scores. Finally, the study applies appropriate multivariate statistical methods and includes regression diagnostics, enhancing the reliability and transparency of the findings.

### 4.8. Limitations

This study has several limitations that should be considered when interpreting the findings. First, the cross-sectional design captures cyberchondria-related behaviors and associated characteristics at a single point in time. Consequently, temporal relationships and causal direction cannot be established, and the observed associations should be interpreted as descriptive rather than explanatory. Second, participants were recruited using a proportionate stratified non-probability convenience sampling approach within a single all-female university. Although proportional allocation across academic programs improved coverage of the participating institution, recruitment within each stratum remained convenience-based. This approach may have introduced selection bias because students who were more available, more interested in health-related topics, or more engaged in online health information seeking may have been more likely to participate. Such self-selection could have resulted in either overestimation or underestimation of cyberchondria-related behaviors within the wider student population. The use of an online survey may also have contributed to differential participation and possible non-response bias.

Third, the study relied on self-report measures. The CSS-12 responses reflect participants’ perceptions and recollections of their own online health-information-seeking behaviors, while GPA was also self-reported. Self-reported data may be affected by recall error, subjective interpretation of questionnaire items, or response tendencies. Therefore, the reported levels of cyberchondria-related behaviors may not fully correspond to objectively observed online-searching behavior or independently verified academic information. Fourth, although the overall analytic sample comprised 362 participants, some academic-program subgroups were relatively small, particularly Dentistry and Pharmacy. These unequal and limited subgroup sizes may have reduced the precision of subgroup comparisons and the ability to detect small between-group differences. Consequently, non-significant findings for academic characteristics should not be interpreted as evidence that no associations exist.

Fifth, several theoretically relevant psychological and digital-behavioral variables, including health anxiety, intolerance of uncertainty, obsessive–compulsive symptoms, eHealth literacy, and problematic internet use, were not assessed. Their omission limited the explanatory capacity of the regression model and may partly account for the very small proportion of variance explained. Therefore, the present findings cannot determine whether these unmeasured factors are associated with cyberchondria-related behaviors in this population. Sixth, the CSS-12 does not have universally established clinical cut-off points. Accordingly, the lower-, middle-, and upper-score categories used in this study were sample-derived descriptive groupings rather than clinically validated categories. These classifications therefore should not be interpreted as diagnostic levels of cyberchondria, and comparisons with studies using different scoring approaches should be made cautiously. Finally, the study was conducted at a single all-female Saudi university. The findings therefore reflect the characteristics of the participating female healthcare students and may not be applicable to male students, students from other universities, non-health disciplines, or populations in different cultural and educational settings. The findings should consequently be regarded as context-specific rather than representative of healthcare students more broadly.

## 5. Conclusions

This study provides context-specific evidence regarding cyberchondria-related behaviors among undergraduate female healthcare students at the participating all-female Saudi university. Based on the CSS-12, participants were distributed across sample-derived lower-, middle-, and upper-score groups. Repeated online symptom searching and consultation of multiple online sources were among the more frequently reported behaviors. These score categories represent descriptive groupings within the present sample and should not be interpreted as clinically validated levels of cyberchondria. Among the CSS-12 dimensions, excessiveness showed the highest mean score, followed by distress, whereas reassurance and compulsion showed comparatively lower mean scores. Significant positive correlations were observed among all four CSS-12 dimensions, indicating that these dimensions were interrelated within the study sample.

The exploratory regression analysis showed that age, GPA, academic year, and academic program were not significantly associated with CSS-12 total scores, and the model explained only a negligible proportion of the observed variance. These findings indicate only that the demographic and academic variables examined in the present model did not meaningfully account for variation in CSS-12 scores within this sample; they should not be interpreted as evidence that such characteristics are unrelated to cyberchondria in other populations or settings.

Given the cross-sectional design, causal and temporal relationships cannot be established. Psychological and digital-behavioral factors, including health anxiety, intolerance of uncertainty, obsessive–compulsive symptoms, eHealth literacy, and problematic internet use, were not assessed and therefore cannot be used to explain the findings of the present study. Future research may examine these variables using theoretically informed multivariable and longitudinal designs to further clarify their associations with cyberchondria-related behaviors. Overall, the findings should be interpreted within the context of the participating female healthcare students at a single all-female Saudi university and should not be considered representative of healthcare students more broadly.

## Figures and Tables

**Table 1:** Frequency and percentage distribution of participants according to their socio-demographic profile (*n* = 362).

Items	*n*	%
Age Group		
18–20	216	59.7
21–23	143	39.5
24–26	2	0.55
27–30	1	0.25
Program		
Foundation Year	66	18.3
Nursing	97	26.8
Health Rehabilitation Sciences	139	38.4
Medicine	28	7.7
Dentistry	12	3.3
Pharmacy	20	5.5
Academic Year		
First	111	30.7
Second	77	21.3
Third	66	18.2
Fourth	72	19.9
Fifth	15	4.1
6th	7	1.9
Interns	14	3.9
GPA		
4.6–5.0	137	37.8
4.1–4.5	158	43.7
3.6–4.0	52	14.4
3.1–3.5	13	3.6
2.6–3.0	1	0.25
<2.5	1	0.25

**Table 2:** Distribution of cyberchondria-related online health information-seeking behaviors among participants (*n* = 362).

Cyberchondria Activities	Mean	SD
Excessiveness Domain		
If I notice an unexplained bodily sensation, I will search for it on the internet.	3.83	1.30
I read different web pages about the same perceived condition.	3.66	0.81
I enter the same symptoms into a web search on more than one occasion.	2.81	1.12
Compulsive Domain		
Researching symptoms or perceived medical conditions online distracts me from reading news/sports/entertainment articles online.	2.79	1.10
Researching symptoms or perceived medical conditions online interrupts my work (e.g., writing emails or working on word documents or spreadsheets).	2.22	0.83
Researching symptoms or perceived medical conditions online interrupts my offline social activities (e.g., reduces time spent with friends/family).	1.97	0.64
Distress Domain		
Start to panic when I read online that a symptom, I have is found in a rare/serious condition.	2.90	1.05
I think I am fine until I read about a serious condition online.	2.59	1.18
I feel more anxious or distressed after researching symptoms or perceived medical conditions online.	1.97	0.78
Reassurance Domain		
Researching symptoms or perceived medical conditions online led me to consult with my GP.	2.90	1.04
I suggest to my GP/medical professional that I Imay need a diagnostic procedure that I read about online (e.g., a biopsy/a specific blood test).	2.25	1.22
Researching symptoms or perceived medical conditions online leads me to consult with other medical specialists (e.g., consultants).	2.58	1.17

**Table 3:** Distribution of CSS-12 score categories across cyberchondria dimensions (tertile-based grouping) (*n* = 362).

Dimension	Upper Score *n* (%)	Middle Score *n* (%)	Lower Score *n* (%)	Min	Max	Mean (SD)
Excessiveness	162 (44.8%)	151 (41.7%)	49 (13.5%)	3	15	10.29 (2.10)
Distress	96 (26.5%)	125 (34.5%)	139 (38.5%)	3	15	7.46 (2.35)
Reassurance	83 (22.9%)	93 (25.7%)	186 (51.4%)	3	15	7.73 (2.20)
Compulsion	54 (14.9%)	88 (24.3%)	219 (60.5%)	3	15	6.98 (2.05)
Total CSS-12 score	86 (23.8%)	110 (30.4%)	166 (45.9%)	12	60	32.46 (5.80)

**Table 4:** Pearson correlation matrix between CSS-12 dimensions.

Variable	Excessiveness	Distress	Reassurance	Compulsion
Excessiveness	1.00	0.42 **	0.31 **	0.45 **
Distress	0.42 **	1.00	0.39 **	0.56 **
Reassurance	0.31 **	0.39 **	1.00	0.52 **
Compulsion	0.45 **	0.56 **	0.52 **	1.00

Note: ** *p* < 0.001 (2-tailed). Values represent Pearson correlation coefficients.

**Table 5:** Multiple Linear Regression Analysis of Factors Associated with CSS-12 Total Score.

Variables	B	SE	β	t	*p*-Value	95% CI	VIF
Constant	32.74	2.31	—	14.17	<0.001	28.20–37.28	—
Age (21–23 vs. 18–20)	0.38	0.57	0.04	0.67	0.503	−0.74–1.50	1.05
GPA	0.11	0.89	0.01	0.12	0.903	−1.63–1.85	1.09
Academic Year (5th year and interns)	−0.94	0.68	−0.08	−1.38	0.168	−2.28–0.40	1.12
Academic Program (Foundation vs. Specialized)	−1.76	1.54	−0.07	−1.14	0.256	−4.79–1.27	1.18

R = 0.077, R^2^ = 0.006, Adjusted R^2^ = −0.005, F-test *p* = 0.712.

## Data Availability

The data presented in this study are available on request from the corresponding author. The data is unavailable publicly unless requested for valid reasons. It was part of the agreement between the researchers and the participants that the data will be used only by the researchers and for the purpose of this specific study.
